# Effects of Soil Water Shortage on Seedling Shoot and Root Growth of Saragolle Lucana Tetraploid Wheat (*Triticum durum* Desf.) Landrace

**DOI:** 10.3390/plants11243492

**Published:** 2022-12-13

**Authors:** Rocco Bochicchio, Rosanna Labella, Roberta Rossi, Michele Perniola, Mariana Amato

**Affiliations:** 1 School of Agriculture, Forestry, Food and Environmental Sciences, University of Basilicata, 85100 Potenza, Italy; 2 Council for Agricultural Research and Economics, Research Centre for Animal Production and Aquaculture (CREA-ZA), 85051 Bella (Potenza), Italy; 3 Dipartimento delle Culture europee e del Mediterraneo, Università della Basilicata, 75100 Matera, Italy

**Keywords:** durum wheat, ancient wheats, root length, rhizosheath, root/shoot ratio

## Abstract

Ancient wheats may be a source of traits that are useful for the tolerance of climate change foreseen conditions of raising temperatures and low water availability. Previous research has shown a fine root system and a high mass of rhizosheath per unit root mass in the italian durum wheat (*Triticum durum* Desf) landrace Saragolle Lucana, and this may be relevant for successfully facing adverse conditions during seedling establishment. We investigated the effect of soil water shortage in Saragolle seedlings on root architecture, rhizosheath formation and biomass allocation. Pot experiments were conducted by comparing two levels of soil available water content (AWC): WW (100% of AWC) and DS (50% of AWC). Phenology was delayed by eight days in DS and above and belowground traits were measured at Zadoks 1.3 for each treatment. Biometric data collected at the same phenological stage show that DS plants did not reach the levels of biomass, surface area and space occupation of WW even after attaining the same developmental stage. Namely, plant dimensions were lower at low soil water availability, with the exception of rhizosheath production: DS yielded a 50% increase in rhizosheath mass and 32% increase in rhizosheath mass per unit root mass. The proportion of plant mass reduction in DS was 29.7% for aboveground parts and 34.7% for roots, while reductions in leaf and root surface areas exceeded 43%. The root/shoot mass and area ratios were not significantly different between treatments, and a higher impact on aboveground than on belowground traits at reduced available water was shown only by a lower ratio of shoot height to root depth in DS than in WW. Increases in rhizosheath in absolute and relative terms, which were observed in our experiment in spite of smaller root systems in the ancient durum wheat variety Saragolle lucana at DS, may provide an interesting trait for plant performance in conditions of low soil water availability both for water-related issue and for other effects on plant nutrition and relations with the rhizosphere.

## 1. Introduction

Climate change foreseen conditions include higher temperatures and lower water availability. Zampieri and co-authors [1] have identified heat and water excess or deficit stress as explaining more than 40% of the inter-annual wheat yield variability at the global level. Research has focused on the effects of water deficit stress on wheat behavior, especially at advanced phenological stages [2] on the grounds that wheat is more sensitive to climatic extremes during the three months period before harvesting [3]. Drought stress during the sensitive stage of anthesis and soil water reserves in post-anthesis are actually among the major yield constraints in the Mediterranean climate. Drought stress tolerance mechanisms, however, start developing as soon as the seminal root system develops, which is at the seedling stage. Early drought stress affects root depth and root area [4] and therefore may contribute to increased root access to deep water reserves, which is critical for wheat growing on stored water. Sanguineti et al. [5] phenotyped seedling seminal root architectural traits in elite durum wheat germplasm and argued that seedling seminal root architecture is very important since these roots remain active throughout the wheat life cycle and grow deeper in the soil profile. Testing drought stress on seedlings is important not only because stress tolerance at this stage affects stand establishment and permanently shape some seminal root traits, but also because plant response to early stress events may play a role in plant response to future drought stress events. Fascinating findings on pea stress memory show that plants keep an imprint of the first stress event, which may improve adaptation toward future stress [6]. In wheat, water and temperature stresses at the seedling stage may affect the timing and conditions of tillering [7], and therefore flowering induction and productive behavior may be severely impaired.

Under the current climate change scenario characterized by rising temperature and prolonged droughts root architecture and the rhizosheath have been identified as key traits for increasing crop resilience and yield stability [2,8].

The length of the longest root and reduction of root diameter correlated well with plant water status in a synthetic hexaploid wheat [9]. In order to be useful in breeding, root traits should also be highly hereditable. Some root morphological traits (root thickness, dry weight, mass and length density) associated with rice drought resistance have shown high values of narrow sense heritability [10]. In double aploid wheat lines, the co-location of QTL of yield component traits (grain weight per spike) and QTL of root traits (length) under drought stress suggest the involvement of root architecture in stress tolerance mechanisms [11]. The rhizosheath is the complex of root-exhuded mucilages and adhering soil, which has been found in different orders of angiosperms [12]. This structure is the object of a growing body of literature and has been identified as a trait for breeding due to its involvement in modifying and stabilizing the soil-pore system and soil-water relations, and in keeping roots hydrated in dry soil [13,14,15]. Soil-plant relations are therefore at the core of rhizosheath research [8]. However, in classical studies, roots growing at low soil water content have been shown to bear a larger rhizosheath [16].

The use of ancient wheat varieties has gained interest and market niches. Grain technological quality of ancient Italian durum wheat landraces derived accessions, such as Saragolla have been analyzed (in comparison with modern cultivars) by Giunta and co-authors [17] and show a lower gluten index. While over time durum wheat breeding has improved yield and technological quality of pasta, shifting from old to modern cultivars has reduced durum versatility [18]. The global concern regarding environmental sustainability of cropping systems, and the current scenario of resource scarcity requires materials with better adaptation to low input systems. Ancient tall durum varieties are very suited for low input systems and marginal lands [19]. Such varieties are also interesting for dual purpose (grain and graze) exploitation [20,21], which is a special form of land sharing that underpins facilitative interaction between livestock and cereal production systems.

In terms of efficiency, ancient wheats are characterized by a low harvest index and low chlorophyll concentration (e.g., [22]), but they are researched as a possible source of traits interesting for low-input conditions, especially regarding plant roots [23,24]. Mediterranean wheat landraces show substantial variability in root traits; these population can be considered a valuable target for breeders, since useful root traits can be introgressed into adapted phenotypes through marker assisted breeding [25]. Significant genetic variability in root traits, namely higher total biomass and both shallow and deep roots weight has been found in bred wheat landraces compared to modern varieties [26]. Root architectural traits of wheat landraces were correlated to yield in drought prone environments: root length, volume and weight as well as gravitropic angle were higher for landraces from the dry areas of eastern Mediterranean Region, and the lowest values were found in accessions from the Balkans [27]. A suit of wheat (durum and landraces) and wild progenitors genotypes from Iran, Syria, Turkey and Lebanon were phenotyped in search of root traits relevant for drought stress resistance: Wild emmer and wild einkorn showed a larger investment in surface layers (more nodal roots), durum landraces allocated more length to the seminal roots compared to wild progenitors; large differences in xylem anatomy were also found that suggest a higher hydraulic conductivity for durum [23]. Seminal root architecture of Italian landrace-derived accessions (Russello SG7, Saragolla and Trinakria) shows that these materials have higher values of primary and total root length but lower root weight than modern varieties; plant height is negatively correlated to root weight and to root-shoot ratio [5]. While shoot/grain traits have been studied, much less is available on roots. Bochicchio et al. [22] have shown that the Italian tetraploid landrace Saragolle lucana has traits pointing to efficiency in acquiring resources in the belowground compartment such as a low root/shoot mass ratio, a fine root system and a high mass of rhizosheath per unit root mass. The response of Saragolle’s root and rhizosheath to soil water has not been studied so far.

We conducted research on Saragolle Lucana seedlings in order to test the hypothesis that water shortage in soil during the establishment phase would affect resource allocation to belowground parts and root architecture and that rhizosheath formation would be enhanced. There is a growing interest in cultivating ancient wheat varieties such as Saragolle Lucana, not only due to the high revenue market niche of traditional breads but also to increase crop systems sustainability, as mentioned. Old, tall varieties are more adapted to low-input cultivation and proved to be especially suitable for dual-purpose management. In such systems, in order to maximize both grain and fodder yield, early sowing is recommended. In southern Italy environments, early sowing would expose seedling to a higher risk of water shortage, in this context it is very important to search the traits linked to better stand establishment under drought stress. Rhizosheath mass (a trait with a moderate heritability) and root system architecture play a key role at these early stages. While more work is needed to evaluate the multi-facet dimension of drought resistance, this is the first contribution of root traits and root structures highly relevant for seed establishment, this information may help agronomists in optimizing the choice of the sowing windows. By providing data on below-ground compartment, this work contributes to fill a gap in the current knowledge of the agronomic traits of Saragolle Lucana, an ancient wheat that may regain more space in future cropping systems.

## 2. Results

### 2.1. Shoot Growth

**Figure 1:**
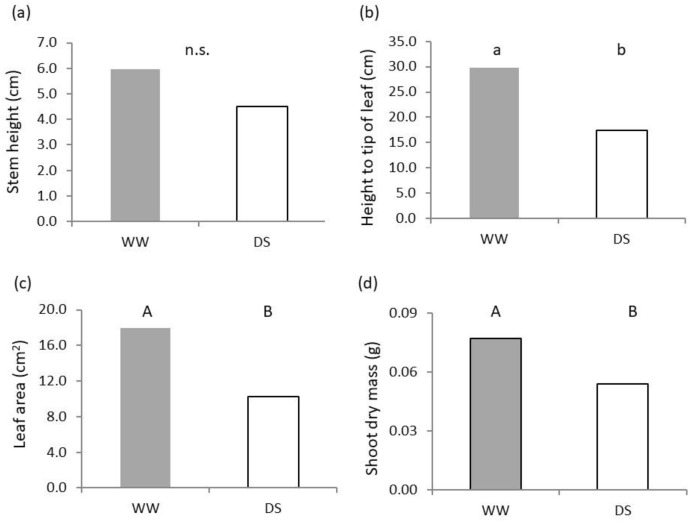
Effect of soil water availability on seedling shoot: (**a**) Height to the uppermost node; (**b**) height to the tip of the uppermost expanded leaf; (**c**) leaf area; and (**d**) shoot dry mass. Different letters on each bar designate significantly different values at the Tukey’s post hoc mean separation test for *p* < 0.05 (lowercase letters), *p* < 0.01 (uppercase letters) or non-significant differences (n.s.) for *p* > 0.05.

The duration of germination and plant establishment up to Zadoks 13 was delayed by eight days at low water availability. Values recorded at equal phenological stage show that plants were taller in the well-watered treatment, but differences did not reach statistical significance for the height of plants at the uppermost node (Figure 1a), whereas height at the tip of the uppermost leaf (Figure 1b) was significantly (*p* < 0.05) lower in the water stressed treatment. In DS plants leaf area was significantly (*p* < 0.01) lower and amounted to only 56.8% of the control (Figure 1c). Leaf length was also significantly different (*p* < 0.01), with values of 29.9 cm for WW and 17.3 cm for DS. Differences in shoot biomass between treatments were significant (*p* < 0.01) as well, and values of DS were 70.3% of those found in WW for dry mass (Figure 1c) and 60.8% for fresh mass (not shown).

**Figure 2:**
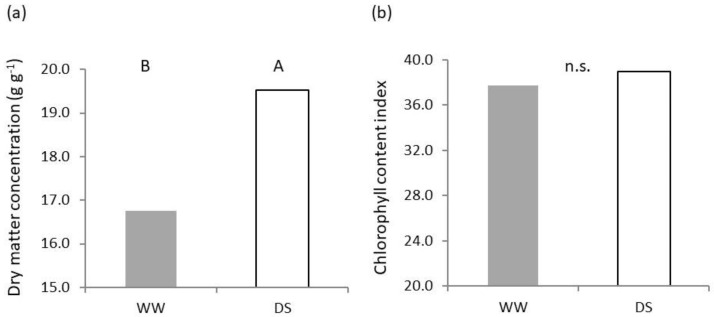
Effect of soil water availability on seedling shoot: (**a**) Dry matter concentration; (**b**) Chlorophyll concentration. Different uppercase letters designate significantly different values at the Tukey’s post hoc mean separation test for *p* < 0.01, or non-significant differences (n.s.) for *p* > 0.05.

Lower water availability resulted in plants with a higher (*p* < 0.01) dry matter concentration (Figure 2a), whereas differences in chlorophyll content were not significant between treatments (Figure 2b).

### 2.2. Belowground Plant Growth and Architecture

**Figure 3:**
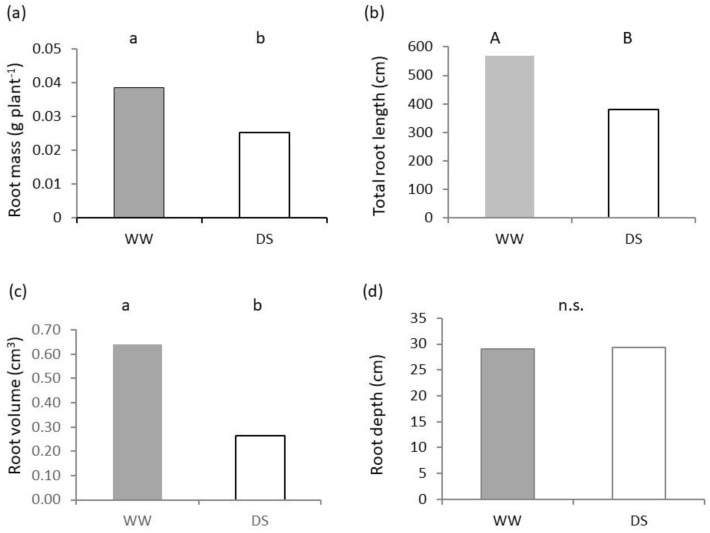
Root growth and architecture of seedlings of Saragolle lucana under different soil water availability. (**a**) Root dry mass; (**b**) total length of root structures; (**c**) total volume of root structures; and (**d**) root depth. Different letters on each bar designate significantly different values at the Tukey’s post hoc mean separation test for *p* < 0.05 (lowercase letters) or *p* < 0.01 (uppercase letters), or non-significant differences (n.s.) for *p* > 0.05.

The mass of roots of Saragolle lucana was reduced by 34.7% in low available water with significantly (*p* < 0.05) lower values than those of WW (Figure 3a). The total length of all root structures recovered from the pots was also significantly (*p* < 0.01) lower in DS (Figure 3b) with a reduction of about one third. The corresponding difference in root volume was 58.9% of the values of well-watered control and was statistically significant (*p* < 0.05) as well (Figure 3c). However, the maximum length of a single root axis (root depth—Figure 3d) was not significantly different.

**Figure 4:**
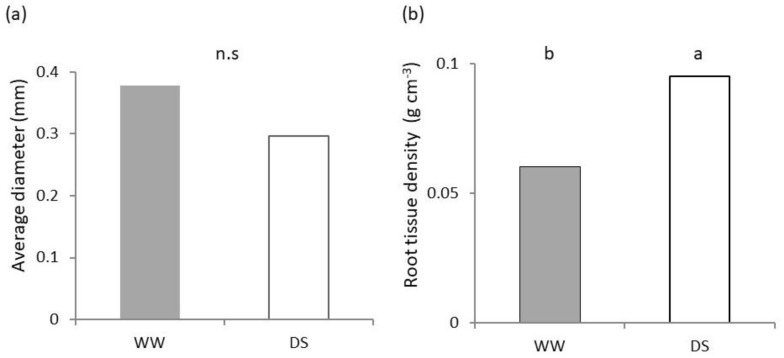
Root architecture of seedlings of Saragolle lucana under different soil water availability. (**a**) Average root diameter; (**b**) root tissue density. Different lowercase letters on each bar designate significantly different values at the Tukey’s post hoc mean separation test for *p* < 0.05, or non-significant differences (n.s.) for *p* > 0.05.

The overall average diameter of roots showed a deviation from normality due to a multimodal distribution. The average value (Figure 4a) was lower in DS, but statistical significance was not found at the non-parametric Kruskal-Wallis test, which yielded a *p* value > 0.05.

The density of root tissues (Figure 4b) was significantly (*p* < 0.05) higher in the DS treatment, which showed an increase of 57.8% compared to the WW values.

**Figure 5:**
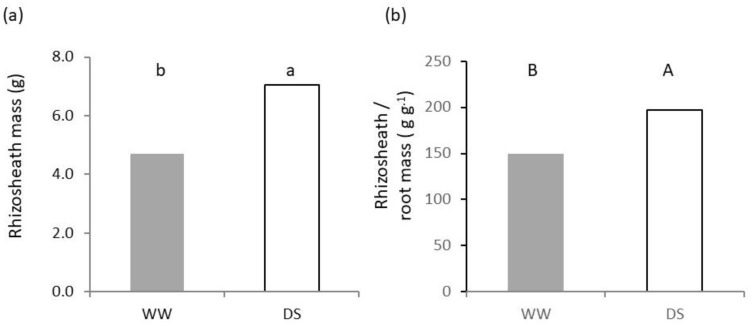
Effect of soil water availability on rhizosheath: (**a**) Rhizosheath dry mass; (**b**) rhizosheath/root dry mass. Different letters on each bar designate significantly different values at the Tukey’s post hoc mean separation test for *p* < 0.05 (lowercase letters) or *p* < 0.01 (uppercase letters).

Data on rhizosheath are shown in Figure 5. A low availability of soil water resulted in a significantly (*p* < 0.05) higher mass of rhizosheath in the seedling roots of Saragolle lucana (Figure 5a). Values recorded in DS were equal to 150% of those found in WW (Figure 5a). The dry mass of rhizosheath ranged between 149.2 g per grams of root mass in WW and 197.0 g g^−1^ in DS (Figure 5b) and differences were statistically significant (*p* < 0.01).

### 2.3. Allometrics

**Figure 6:**
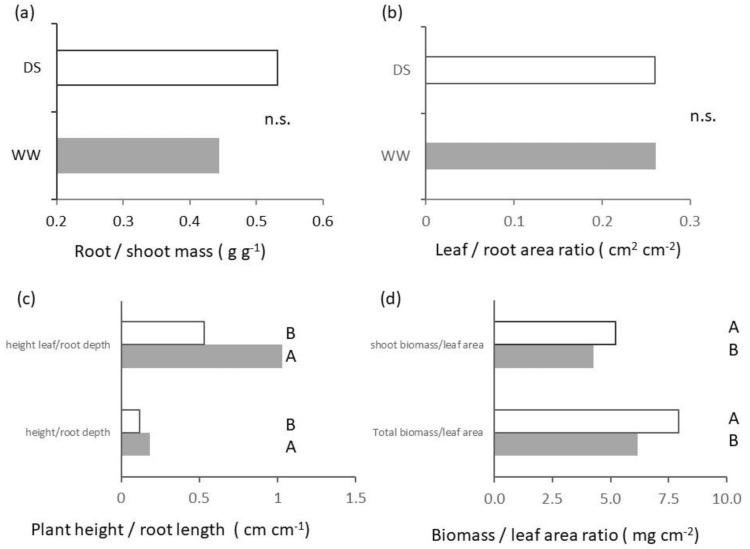
Effect of soil water availability on allometrics. (**a**) Root-to-shoot dry mass ratio; (**b**) biomass-to-leaf area ratio; (**c**) shoot height-to-root depth ratio; and (**d**) leaf-to-root area ratio. White bars: DS; grey bars: WW. Different letters on each bar designate significantly different values at the Tukey’s post hoc mean separation test for *p* < 0.05 (lowercase letters) or *p* < 0.01 (uppercase letters), or non-significant differences (n.s.) for *p* > 0.05.

Figure 6 reports indices calculated from the ratios of biometric variables relative to above and/or belowground plant traits. On average seedlings of Saragolle allocated 0.44 g of dry matter to roots per gram of dry matter found in shoots for the well-watered control and 0.53 g g^−1^ in the soil with lower water availability (root/shoot ratio Figure 6a) but differences did not reach statistical significance. Allocation of surface areas was in favor of the below-ground compartment since, for each square centimeter of root area calculated with image analysis, the area of leaves was on average 0.26 cm^2^ without significant differences between treatments (Figure 6b). The ratio of shoot height to root depth potential (maximum length of a root axis) is reported in Figure 6c. It shows significantly (*p* < 0.01) higher values in WW than in DS both for height to the uppermost node and height to the tip of the uppermost leaf. The dry biomass acquired per unit leaf surface area (Figure 6d) were significantly (*p* < 0.01) higher for seedlings grown with low available water. Values of DS were 22.2% higher than those of WW for shoot biomass and 28.6% for total plant dry mass with a maximum of 7.9 mg of dry mass acquired for every cm^2^ of leaf area.

## 3. Discussion

Our results show the effects of reducing soil water availability to 50% on phenology, quantitative growth variables and on their relations in ancient durum wheat cultivar Saragolle lucana before tillering.

The moderate drought stress level chosen in this study provides a first stress threshold for Saragolle, the drought sensitive threshold shows substantial genetic variability in wheat seedlings [28]. The choice of testing plant ability to withstand a moderate rather than an extreme drought stress stems from the necessity to mimic realistic field conditions. Passioura [29] argues that extreme droughts are rare in natural fields and even if breeding efforts could increase plant survival to severe drying this does not imply increasing crop productivity; selecting traits(genotypes) that are capable of surviving to extreme drought events may be more relevant for perennials. Increasing plant ability to withstand moderate (which is likely to be the average) rather than extreme drought is a promising breeding goal for annuals such as wheat.

Phenology during wheat establishment is important for the timing of sowing: Wheat tolerance of low temperatures is the maximum during tillering [7] and vernalization needs require that tillering plants undergo a sufficiently long cold period for winter-types, and this may be combined with photoperiod sensitivity [28,30]. Sowing is therefore scheduled at each latitude and altitude in order to ensure that such a stage is synchronized with seasons and/or temperature cycles. Our data show that, in spite of the potential effects of higher fall temperatures on a faster germination [7], a contemporary drought stress may delay phenology and the two contrasting effects need to be further studied for tuning sowing schedules in view of climate changes. Additionally, in our experiment, biometric data collected at the same phenological stage show that DS plants did not reach the levels of biomass and space occupation (leaf area, root length, etc.) of WW even after reaching the same level of development. Therefore, growth reductions at low water availability cannot be entirely ascribed to delays in phenology and are not compensated for by a longer growing time. Consistent with our results, a moderate drought stress (deficit irrigation at 50% FC) halved shoot biomass (reducing more fresh than dry biomass) and leaf area in Spartina alterniflora (Poaceae) a native perennial C4 used as fodder source in arid and saline environments of Tunisia, similarly to our work the shoot was more sensitive to drought stress than roots [31]. Deficit irrigation at 50% FC was also sufficient to drastically reduce shoot growth in the dicot damask rose [32] in this experiment plants were able to withstand a moderate drought stress, also through osmotic adjustments, which was ineffective at 25% of the FC stress level. Thus, Saragolle proves to be sensitive to drought stress at least during the juvenile stage.

Leaf chlorophyll content was slightly higher, but non-significantly different under drought stress, in a similar setup (50% vs. 100% field capacity) leaf nitrogen content increased by 19% in drought stressed Spartina alterniflora a native C4 graminoid of arid and saline Mediterranean marshes [33]. In drought stressed leaf nitrogen increased along with the proline and soluble carbohydrates pools which increased the leaf osmotic potential. Osmotic adjustment, which is a key mechanism for drought and salinity stress tolerance [32] remains to be tested in Saragolle; our data indicate so far that Saragolle shows a marked allometric response to drought stress to maintain leaf turgor, no signs of wilting were detected at the end of the experiment. Drought can cause chlorophyll breakdown by damaging chloroplasts [33]. As leaf chlorophyll content respond to drought stress SPAD has been proposed as a physiological indicator of the photosynthetic machine integrity and a rapid screening tool for drought tolerance [34]. Physiology in regard to leaf chlorophyll content was not impaired at this stress level as SPAD in stressed plants did not change, showing rather slightly higher values than the well-watered control, if this was the result of an osmotic adjustment/chlorophyll protective mechanism, a consequence of biomass and photosynthetic adjustment or both remains to be ascertained.

Root architecture plays a key role in resource acquisition efficiency; useful root traits under drought stress such as a narrow root angle [35], higher specific root length and high root-shoot ratio are related to space occupancy, metabolic costs of root exploration and foraging capacity. In our research, above and below-ground dimensions of seedlings at 50% available water content were in general lower than that of the 100% AWC control measured at the same phenological stage of Zadoks 1.3 (Figure 1 and Figure 3). Low water availability is often reported to cause a lower carbon fixation due to lower stomatal conductance and non-stomatal effects known as metabolic inhibition [36], but in many cases carbon partitioning is also affected and a higher proportional reduction in shoot allocation is recorded. As a result, a higher root/shoot ratio is found in the literature under water deficit stress (e.g., [37]) up to 50% in wheat seedlings [38]. Allometric indexes based on root and shoot area (i.e., ratio of root area to leaf area, or root length to leaf area), can be considered proxies of the proportion of absorbing to transpiring plant surfaces, and are more predictive of root functioning than indexes based on mass fractions [39]. In our data we were unable to prove a different proportion of growth reduction between above and belowground plant parts, as shown by lack of significance of differences in mass and area ratios of shoots and roots (Figure 6a,b). Nevertheless, a significant stronger proportional effect in impairment of shoots at 50% AWC is shown by the ratio of shoot height to root depth (Figure 6c), an index of relative potential of space occupation by plants in the aerial vs. soil environment. Values were lower for DS and this indicates a lower investment in aboveground space occupation than in belowground at reduced available water.

Allometric relations in plants are a function of genetics, environmental conditions (e.g., [40]) and interactions with micro-organisms (e.g., [22]). The percent fraction of above and belowground portions varies by balancing functions of resource acquisition and consumption of each part. Of our allometric indicators, the ratio of shoot height to root depth would support classical theories of functional balance [41] where the dimensions of above and belowground parts reflect the trade-offs of carbon fixation and water loss by leaves as in equilibrium with water and nutrient uptake and carbon use of roots. Within this framework a lower soil water availability would result in a higher proportional investment in roots in order to face unit shoot water requirements. Wang and co-authors [42] have challenged this view by showing the role of root architecture changes (diameter, surface, etc.) in modulating resource acquisition in response to environmental conditions.

Root biometric values are highly variable with the genotype and environment. Even in uniform conditions within a phenotyping platform, Chen and co-authors [43] report a more than four- and five-fold difference for root length and dry mass respectively among 184 genotypes at the onset of tillering. In their study, diameters ranged from 0.26 to 0.48 mm and root tissue density between 75.4 and 175 g cm^−3^. For the same variety as that of our experiment, Bochicchio and co-authors [22] report a fine root system with a small average diameter (around 0.29 mm for seedlings) but no significant diameter variation in response to inoculation with *Trichoderma harzianum* T-22. In our data (Figure 4a), the overall average diameter of DS was lower than that reported in [22] and that of WW was higher. The plurimodal nature of diameters due to different root classes, though, resulted in a lack of statistical significance of differences between out treatments even with a nonparametric test. A fine root system is identified as exhibiting high potential for maintaining productivity under drought [39] and its role in fast water uptake in conditions of low precipitation has been linked to the high water-spending behavior of ancient wheats [11].

The classical theory of resource economics spectrum classifies roots into fine (low diameter) systems which maximize resource acquisition and thick (high diameter and high tissue density) systems which maximize resource conservation [44]. However, recent evidence questions such a framework by showing a negative correlation of root tissue density with root diameter (e.g., [36]). In our case, in spite of tendentially (though not significantly) lower root diameter plant tissues had a higher density both in the shoot (Figure 6d) and root (Figure 4b) compartment in DS. A higher biomass per unit leaf area recorded at low AWC (Figure 6d) was not matched by differences in chlorophyll content (Figure 2b); it is likely rather an index of lower intercellular spaces and/or high resource storage.

Our work reports variation in rhizosheath (Figure 5) in response to water availability.

Delahaizeet and co-authors [45] have shown that wheat rhizosheath traits are genetically controlled, therefore they represent a good target for breeding. Bochicchio et al. [22] quantified the rhizosheath of Italian durum wheat varieties released at different times and found large differences in rhizosheath mass; in that work Saragolle lucana showed the highest value of rhizosheath per unit root mass. Our data report a rhizosheath mass about four times higher than those of [22], in spite of similar root and shoot growth mass data. This discrepancy is linked to differences in methods used for separation of bulk from rhizosheath soil: in our work we extracted the rhizosheath-root complex by gently shaking off bulk soil, whereas [22] in addition to shaking also brushed off soil that was not strongly attached and thus excluded the part of rhizosheath that was weakly bound. Methods for the quantification of rhizosheath are not standardized, nor has the strength of root-mucilage-soil links been defined in unambiguous physical or operational terms. The rhizosheath of cereals has been reported to be bound more strongly than for legumes [8] so that different techniques are applied. In our case, we choose to include all components of the rhizosheath.

At low available soil water, the mass of rhizosheath was significantly higher than in WW by 50% (Figure 5a) and was equal to about 149 to 197 times the mass of roots in WW and DS, respectively (Figure 5b). Bochicchio and co-authors [22] found that inoculation with *Trichoderma harzianum* T-22 increased the amount of rhizosheath from 9.4% to 36.1% in different wheat varieties, including Saragolle lucana, and proposed that some of the improvements of plant growth and health observed upon application of *Trichoderma*–based biostimulants may in fact be due to effects on the rhizosheath enhancement. In general, though, rhizosheath is a trait connected to plants from deserts and dry environments [14] and is classically reported to be enhanced at low soil water [16]. However, mechanisms of root-soil interactions related to water uptake are controversial and range from the role of rhizosheath mucilages in keeping root hydration and soil-root contact, to altering pore geometry [15] and structure stability [46]. Additionally, an important role is played by slower rewetting of rhizosheath after drying and changes in the soil water surface tension [47]. In some cases, this has been associated with a higher degree of transpiration in wheat [14].

Rabbi et al. [8] found different cover and mass of rhizosheath in eight wheat cultivars and showed that this trait was linked to transpiration, exudation of metabolites affecting water surface tension and relations with bacterial abundance. The rhizosheath of wheat has also been proven to be involved in processes linked to phosphorus, iron, sulphur and aluminium [48,49] availability. Therefore, increases in rhizosheath in absolute and relative terms, which were observed in our experiment in spite of smaller root systems in the ancient durum wheat variety Saragolle lucana at DS, may provide an interesting trait for plant performance in conditions of low soil water availability both for water-related issue and for other effects on plant nutrition and relations with the rhizosphere.

Our data show that, in spite of potential effects of higher temperatures on faster germination, a contemporary low soil-water availability may delay phenology, and the two contrasting effects need to be further studied for tuning sowing schedules in view of climate changes. Additionally, growth reductions at low water availability cannot be entirely ascribed to delays in phenology and are not compensated for by a longer growing time.

Saragolle is an ancient wheat and it has been acknowledged that wheat landraces are a valuable source of genetic variability for increasing drought tolerance [50]. The selective pressure for Saragolle lucana was driven by the local environment, in terms of water availability Basilicata wheat belt ranges from low to medium high rainfall availability thus a growing environment predominantly characterized by moderate but not extreme droughts. Not much is known about ancient wheat varieties above and below-ground traits, but studies comparing rooting depth of modern and old wheat showed that old varieties had deeper roots only in well-watered conditions while under drought stress modern wheat enhanced root proliferation in deep soil [51].

Mejri et al. [52] compared seedling resistance to transient drought stress in cultivated and wild barley for one, two, three and four weeks, although no direct comparison can be done due to the difference between experiments in drought stress intensity (higher than our), management (transient water withholding vs. deficit irrigation) and timing (60 days old vs. 14 days old seedlings) the magnitude of shoot biomass reduction we obtained is more similar to the response of cultivated than wild barley after a drought period of two weeks, wild barley however showed an outstanding recovering capacity when re-watered. Our data demonstrate that Saragolle is quite sensitive to a moderate early drought stress, however since the photosynthetic machine was not damaged full recovery could be expected. Mejri et al. [52] highlight an interesting issue in drought stress experiments, in nature stress is often transient and rarely kills the plant therefore besides resistance(survival) to a single stress event a key trait worth investigating for increasing stress tolerance is crop resilience. Plant stress memory, which enables the plant to increase its tolerance toward future stress events is a multi-facet process, a thorough investigation of drought resistance requires a whole-cycle evaluation [53]. For Saragolle, an ancient cultivar with a brilliant future in the context of achieving more sustainable cropping systems [21], forthcoming work may address the uncovered issues of the physiological (metabolic) pattern of drought resistance and plant recovery throughout multiple stress events.

## 4. Materials and Methods

Experiments were conducted on the Italian ancient durum wheat landrace Saragolle Lucana (Italian Wheat Landrace Conservation Registry https://www.gazzettaufficiale.it/eli/gu/2014/01/28/22/sg/pdf, accessed on 12 December 2022).

### 4.1. Available Water Treatments

Two treatments with 6 replications were set on the basis of soil water content in pots. Available water content (AWC) was determined as the difference between water retained at −0.033 MPa (defined as field capacity) and water retained at −1.5 MPa (defined as permanent wilting point). Treatments were:

(i) Well-watered control (WW) where potting soil was mixed with water in order to bring it to field capacity. This corresponds to filling pots to 100% of AWC corresponding to 47.8% of total porosity.
(ii) Low available water for water deficit stress (DS) where potting soil was mixed with water in order to bring it to 50% of AWC corresponding to 23.9 % of total porosity.

### 4.2. Seedlings Growth Conditions

Seeds were selected for weight uniformity (average weight 54.22 mg Std.dev 0.69 mg) Seeds were pregerminated on filter paper with distilled water for 48 h and then planted in pots.

Pots were cylinders of 25 cm length and 2.8 cm diameter and were filled up to the hiught of 23 cm with 155.0 g of a field collected silty loam soil with the following characteristics: Sand (50–2000 μm) 43.6%, silt (2–50 μm) 34.2%, clay < 2 μm) 22.1%., and pH 6.8; N 1.9 g kg^−1^; phosphates (P_2_O_5_) 50.3 g kg^−1^; potassium oxide (K_2_O) 1430 g kg^−1^. Pots were arranged randomly in a custom-built growth cabinet and grown until the 3rd leaf was fully expanded (Zadoks 13) [54] at average T = 26.4 °C; average relative humidity 46.06% to simulate germination in high-temperature conditions. Pots were irrigated with 2 mL every 3 days in order to simulate night condensation during emergence without refilling the soil profile.

### 4.3. Measurements

At the end of the experiment the following measurements were made:

Chlorophyll content index (CCI) on three leaves per plant with a leaf transmittance leaf clip chlorophyll concentration meter (MC-100 Apogee instruments Logan, UT-USA):

CCI = T_931_÷T_653_

where T_931_ = Leaf transmittance at 931 nm; T_653_ = Leaf transmittance at 653 nm. Average CCI per plant was then calculated.

Biometric measurements: plant height to the uppermost internode and to the tip of the last fully expanded leaf were measured. Plants were clipped at the soil level. Leaves were cut at the node and scanned by STD 4800 Image Acquisition Systemat 1200 DPI. Leaf area was then measured on scanned images using WinRhizo ArabidopsisV2009c image analysis software (Regent Instruments Inc., Sainte-foy, QC, Canada).

Above-ground plant fresh and dry (after oven drying at 70 °C until constant weight) biomass was determined on clipped plants.

**Figure 7:**
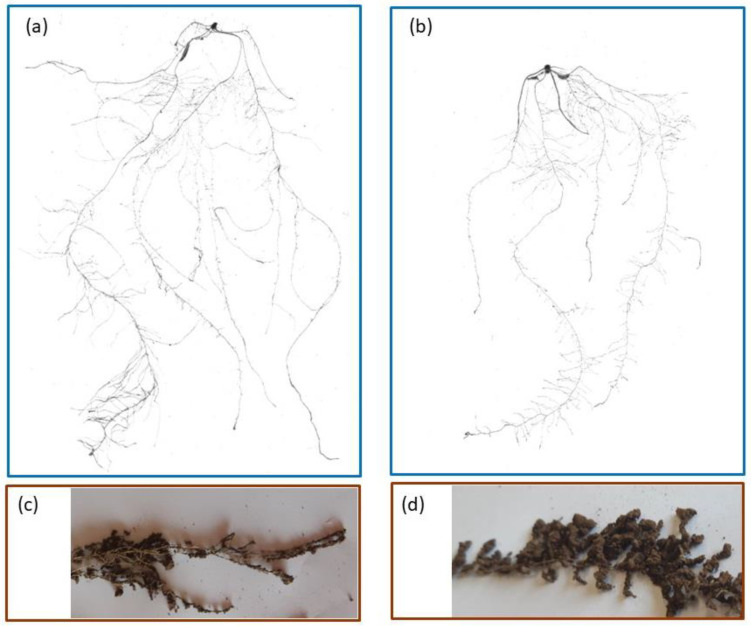
Images of belowground parts of sample plants: (**a**) Scanned image of roots of one sample plant at WW; (**b**) scanned image of roots of one sample plant at DS; (**c**) root with rhizosheath at WW; and (**d**) root with rhizosheath at DS.

The bottom of the pots were removed and the soil was gently pushed from the bottom. On three replications, the root system was extracted by washing over a mesh of 0.5 mm and placed in a transparent tray (200 × 250 mm) with a 4 mm to 5 mm deep layer of water and scanned by STD 4800 Image Acquisition Systemat 600 DPI. After scanning roots were blotted and weighed to obtain the fresh root mass, then oven dried at 70 °C until constant weight to obtain the root dry mass. Root morphology was determined with the same image analysis software mentioned for leaf area. The following traits were measured: Total length (cm), surface area (cm^2^), mean diameter (mm), and volume (cm^3^). The root depth potential was measured as the maximum length of a root axis. All parameters were assessed on a plant basis. Figure 7 reports the scanned images of one sample plant for the WW (Figure 7a) and DS (Figure 7b) treatments each.

We then calculated the root to shoot biomass ratio (g g^−1^), root depth to plant height ratio (cm cm^−1^), leaf-to-root area ratio (cm^2^ cm^−2^), root mass to volume ratio (root tissue density g cm^−3^), biomass-to-leaf area ratio (g cm^−2^), and shoot height-to-root depth ratio (cm cm^−1^).

On three replications, the mass of rhizosheat soil was determined as follows: The root system was held by the plant basis and gently shaken free from bulk. The soil which remained attached to roots after shaking was considered rhizosheath soil. Figure 7c,d show photographs of the rhizosheath for the WW and DS treatments, respectively. The rhizosheath-root complex was then weighed to determine the fresh mass (RRhizFM g plant^−1^), oven-dried at 105 °C and weighed again to obtain the dry mass (RRhizDM g plant^−1^). Roots were thereafter washed over a 0.5 mm mesh, blotted and weighed to obtain the fresh root mass (RFM g plant^−1^), then oven dried at 70 °C until constant weight to obtain the root dry mass (RDM g plant^−1^). The rhizosheath soil fresh (RhizFM) and dry (RhizDM) mass were then calculated as:

RhizFM = RRhizFM − RFM   g plant^−1^

and

RhizDM = RRhizDM − RDM  g plant^−1^

We then calculated the percent increase of RhizDM following inoculation (RhizDMincr) as:

RhizDMincr = 100 × (RhizDM of inoculated plants − RhizDM of control plants)/RhizDm of control plants.

As a consequence of the described procedures, we had 6 replicated for the following variables: Plant height, shoot dry mass, average CCI, root dry mass, and derived indices; the number of replicates was 3 for root length, surface area, diameter, volume, RhizFM, RhizDM, RhizDMincr and derived indices.

Statistical analysis was performed using the R programming language (R Development Core Team, 2020, Vienna, Austria). Homogeneity of variance and normality were tested, respectively, with the tests by Bartlett and Shapiro with a *p* value of 0.05. Where the hypothesis of normality/homogeneity was rejected, the Kruskal-Wallis nonparametric test was used to assess the significance of differences. For all other variables, one-Way ANOVA was performed.

## Data Availability

The data presented in this study are available on request from the corresponding author.
